# Adolescents’ self perceived acne-related beliefs: from myth to science^☆^^☆☆^

**DOI:** 10.1016/j.abd.2019.02.005

**Published:** 2019-10-26

**Authors:** Milica Markovic, Ivan Soldatovic, Milan Bjekic, Sandra Sipetic-Grujicic

**Affiliations:** a Department for Facial Dermatoses, City Institute for Skin and Venereal Diseases, Belgrade, Serbia; b Institute for Medical Statistics and Informatics, Faculty of Medicine, University of Belgrade, Belgrade, Serbia; c Department of General Dermatovenereology, City Institute for Skin and Venereal Diseases, Belgrade, Serbia; d Institute of Epidemiology, Faculty of Medicine, University of Belgrade, Belgrade, Serbia

**Keywords:** Acne vulgaris, Adolescent, Perception

## Abstract

**Background:**

Acne vulgaris is recognized as the third most prevalent skin disease worldwide, with highest prevalence among adolescents. Beliefs and perceptions of adolescents about acne are incoherent in the literature.

**Objectives:**

To assess the most frequently reported acne-related beliefs in adolescents in order to focus on misconceptions and develop proper recommendations.

**Methods:**

A cross-sectional community-based study on representative sample of 2516 schoolchildren was conducted in six randomly selected secondary schools in Belgrade, Serbia. Only schoolchildren with present or past acne history participated. Factors believed to aggravate or ameliorate acne were recorded and analyzed, and the comparisons between girls and boys were evaluated using Pearson's chi-squared test.

**Results:**

A total of 1452 schoolchildren with acne participated, aged 14–18 years, among them 801 (55.2%) girls and 651 (44.8%) boys. Boys significantly more frequently believed that sweating, exercise, and dairy foods aggravate acne, whereas girls significantly more frequently blamed emotional stress, sweets, fatty foods, sun, and lack of sleep. The top four amelioration factors were as follows: comedone extraction, healthy diet, sun exposure, and increased water consumption. Acne regression was more frequently perceived to be linked with cigarettes in boys, but with sun exposure and weight loss in girls.

**Study limitations:**

The narrow age span of adolescents (14–18 years) and exclusion of acne-free adolescents are limitations due to study design.

**Conclusion:**

This survey is part of the first epidemiological study on a representative sample in the Western Balkan region. The significance of the most frequent acne-related beliefs is discussed and myths about acne are highlighted.

## Introduction

Acne vulgaris is recognized as an almost universal cutaneous disease, the third most prevalent skin disease worldwide, with highest prevalence among adolescents, ranging between 40% and 70%.^1^ Apart from a few small-scale studies focused on quality of life issues, and one Croatian study based on knowledge gaps in acne patients and physicians, basic epidemiological studies on representative samples of adolescents with acne in the countries of the Western Balkans have not been performed yet.^2^, ^3^, ^4^ Studies based on beliefs and perceptions regarding acne have involved either only acne patients/adolescents^5^, ^6^, ^7^, ^8^, ^9^ or all students/schoolchildren in the community setting^10^, ^11^, ^12^, ^13^; the latter are purposely conducted to assess the general knowledge on acne and treatment-seeking behavior. Their idea is mainly to target the specific vulnerable population with tailor-made educational programs by clarifying the myths and misconceptions about acne. The present research is a community-based study on a representative sample of adolescents with self-evaluated acne; their beliefs about possible factors that may contribute to acne aggravation or amelioration are analyzed. The idea is to summarize, classify, discuss, and correlate those factors with the objective, evidence-based background of each specific reported belief. Moreover, the similarities and differences in acne-related beliefs between adolescents here in the Western Balkan region and worldwide are somewhat expected and are also included in study design, but represent the secondary aim of the survey. The primary goal is to compare the most frequently reported beliefs of these adolescents with available evidence-based literature in order to focus on misconceptions and acquire proper recommendations for health-related behavior in adolescents with acne of this region.

## Methods

The wider protocol of present study was prepared in collaboration with the Institute of epidemiology, University of Belgrade, and the survey encompassed acne-related aspects of epidemiology, risk factors, quality of life assessment, therapy-seeking behavior, and beliefs and perceptions of schoolchildren in Belgrade, Serbia.

### Participants

The ethics committee of the Faculty of Medicine of the University of Belgrade approved the proposed study design. A cross-sectional survey of schoolchildren aged 14–18 years was carried out during three consecutive winter months, from December 2012 to February 2013. All schoolchildren attending six randomly selected secondary schools in Belgrade were invited through school and parents’ boards, as well as personally by the research team during the initial phase. When adjusted for prevalence rates of adolescent acne of approximately 40–70% and a suggested response rate of 80%, the representative sample of surveyed pupils equaled approximately 2500 children. Written consents from both parents and children were mandatory and the enrollment for study was on voluntary basis.

In order to select participants for the present study, this study engaged only schoolchildren with present and/or past acne history, using the criterion “have you ever had acne?” Schoolchildren with negative answers (“never”) were excluded and only the “acne” group participated.

### Questionnaires and assessment of acne

Selected questionnaire included only acne-related questions – present and past history of acne, and perceived acne triggering and aggravating factors. All questions about perceived factors were designed with three possible answers: “yes,” “no,” and “not exposed.” When further analyzed, “not exposed” were excluded, so that “yes” and “no” answers equaled 100% for each factor. A total of 16 questions for aggravating and eight for ameliorating factors were included.

### Statistics

For descriptive purposes data were presented as numbers with percentages. The categorical variables were described using frequency charts. Comparisons between girls and boys were evaluated using Pearson's chi-squared test with continuity correction. The adopted significance level was 5%. Significant values (*p* < 0.05; *p* < 0.01; *p* < 0.001) were listed in the footnotes of the figures.

## Results

Schoolchildren in all six schools were invited (2833 pupils); of them, 2521 were willing to participate (89% response rate). However, during further evaluation 14 questionnaires were missing, thus the final sample size was 2516 pupils. The majority were between 15 and 17 years of age, and about one-third (29.7%) of pupils were 16 years of age.

For the purposes of the present study, a total of 1452 schoolchildren (57.7% of all participants) with present/past history of acne were enrolled, among them 801 (55.2%) girls and 651 (44.8%) boys.

### Perceived factors that aggravate/ameliorate acne

**Figure 1:**
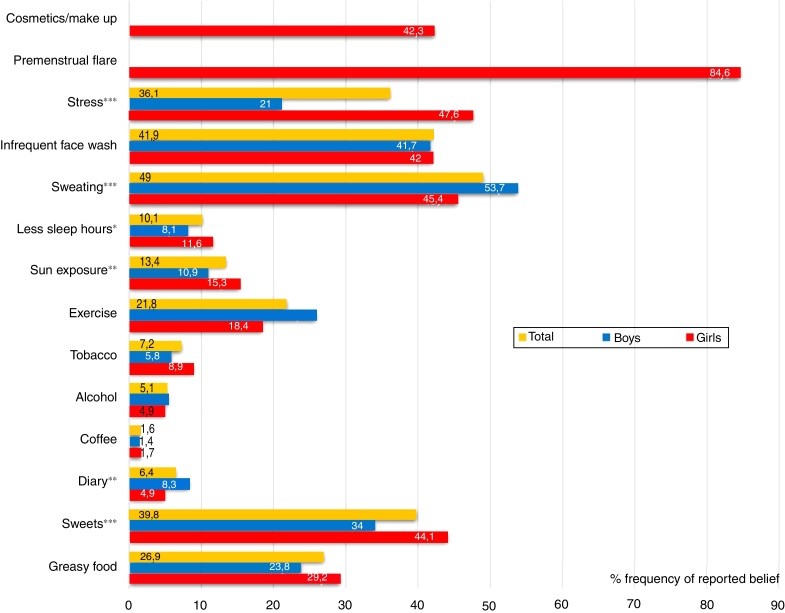
Sex-based differences in self-perceived factors which aggravate acne among schoolchildren with acne (*n* = 1755). Mean values for total boys and girls are given in both columns and numbers (percentages). The chi-squared test was used to determine sex-based differences in reported frequencies, labeled with *p < 0.05; **p < 0.01; ***p < 0.001.

The top four factors believed to aggravate acne in all surveyed pupils with acne were as follows: excessive sweating, infrequent face washing, consumption of sweets, and emotional stress; in all categories except for face washing, gender oriented answers had highly significant differences, as presented in Fig. 1. In general, boys significantly more frequently believed that sweating (53.7%), exercise (25.9%), and dairy foods (8.3%) aggravate acne, whereas girls significantly more frequently blamed emotional stress (47.6%), sweets (44.1%), fatty foods (29.2%), sun (15.3%), and lack of sleep (11.6%) for acne worsening. A selected female-oriented question revealed that premenstrual flare is recorded in 84.6% of girls with acne, and in a question about “cosmetics/make up use” that was answered only by girls, 42.3% believed that regular use of make-up and beautifying skincare aggravate their acne.

**Figure 2:**
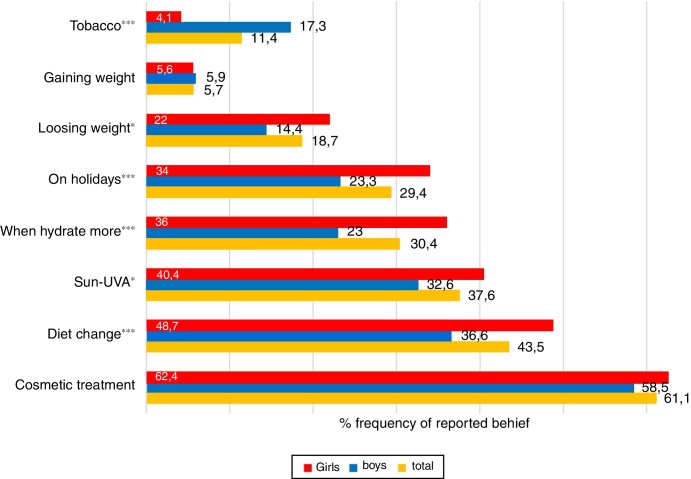
Sex-based differences in self-perceived factors which ameliorate acne among schoolchildren with acne (*n* = 1755). Mean values for total boys and girls are given in both columns and numbers (percentages). The chi-squared test was used to determine sex-based differences in reported frequencies, labeled with *p < 0.05; ***p < 0.001.

The frequencies of reported acne amelioration factors are presented in Fig. 2. The majority of pupils believed in the benefits of comedone extraction, *i.e*., acne treatment led by a nurse practitioner, with no sex-based difference. The other most prevalent overall reports on ameliorating factors were as follows, according to decreasing frequency: diet change to healthier behavior, ultraviolet-A/sun exposure, and increased water consumption (“when hydrated more”). Girls and boys shared this order of frequencies, although boys equally frequently demonstrated that water consumption (23%) and being out of school on holidays (23.3%) ameliorated their acne, the latter was reported by 34% of girls as well. Boys also more frequently assumed that cigarettes could ameliorate their acne (17.3%). In turn, girls were more convinced of beneficial effects of exposure to sun/UVA (40.4%) and losing weight (22%) on their acne.

### Study limitations

One important limitation is the age span of adolescents, which was limited to high school pupils of 14–18 years old, thus early and late adolescence are not included. Moreover, beliefs on acne ameliorating/aggravating factor were collected only in adolescents with acne, in order to target their specific misconceptions; the opinions of acne-free adolescents were not included.

## Discussion

Adolescents surveyed in Greece, Turkey, and Western Europe believe that exacerbation factors of acne rely on diet, inappropriate hygiene, and hormonal changes; they then subsequently blame stress and infection, and lastly cosmetics/make up and sweating.^9^, ^12^, ^13^, ^14^ The analysis of acne exacerbation factors in the present study revealed that nearly half of all female respondents (47.6%) and slightly more than 1/3 of all participants believed that emotional stress triggers acne. Their bidirectional relationship is evidenced; stress might well be the consequence of acne,^15^ but also stress and anxiety caused by life events may aggravate acne, especially inflammatory lesions in males.^16^, ^17^ There is substantial evidence about stress-related neuroendocrine regulation of the sebaceous glands and its role in the pathogenesis of acne.^18^

However, slightly more than half of the male respondents in this study blamed sweating and one-quarter of them blamed exercise for acne exacerbation, which has been reported in similar studies on adolescents and young adults.^19^, ^20^ Sweating itself isn’t involved in the etiopathogenic cascade of truncal acne, but the circumstances in which sweating occurs, such as humidity, prolonged maceration of the stratum corneum, and occlusion by clothes may contribute to occlusion of the pilosebaceous ducts. Nonetheless, a randomized pilot study on males failed to obtain the expected worsening effect of sweating on truncal acne.^21^ The evidence regarding excessive sweating and acne are lacking and remain inconsistent.

The evidence for the role of improper or insufficient facial hygiene in acne pathogenesis is mostly of poor quality.^14^ Nevertheless, infrequent face washing, as the second most commonly reported aggravating factor in this study, is also firmly established in literature reports worldwide.^5^, ^13^, ^22^, ^23^ When analyzing acne and control subjects, Ghodsi et al.^24^ did not demonstrate a difference between those groups in terms of frequency of face washings or use of cleansers/soaps instead of clear water. Still, there is evidence that in those with acne, washing with cleanser twice daily is an appropriate measure for better acne clearance with no irritation.^25^

Despite the fact that the relation of acne with diet was largely considered as a myth, a new era of research at the beginning of the millennium provided a revised and more critical viewpoint, and debate regarding the exact nutrients that influence acne pathogenesis is ongoing.^26^, ^27^, ^28^ Nonetheless, food-related acne beliefs have remained unchanged, and have not been influenced by general dermatologic guidelines.^29^, ^30^ In the present study, the most reported consumables/factors were the following, in decreasing order: dietary change, sweets, increased water consumption, fatty foods, soft drinks, and dairy. Sweets, fatty foods, and change in dietary habits towards healthier food were statistically more frequently reported by girls compared to boys, similarly as reported among Greek adolescents.^9^ The acnegenic properties of both hyperinsulinemic foods and consumption of dairy proteins, which increase concentrations of insulin and insulin-like growth factor (IGF-1), have been proposed by Melnik et al.^31^ Although Kaymak et al.^32^ did not observe correlation of acne with serum glucose and insulin, several other studies conducted by groups of authors^30^, ^33^, ^34^ revealed that the dietary glycemic index (GI), saturated fat, trans-fat, and milk may influence or aggravate acne, and the role of milk is further acknowledged by the Italian group.^35^ In the present study, dairy products were not recognized as a significant acne-aggravating factor. In contrast, weight loss was recognized as a ameliorating factor among almost one-fifth of participants. Although certain studies^20^, ^35^ have demonstrated that increased body mass index (BMI) is described as an acne risk factor and there is evidence that increased BMI is correlated with acne in males,^36^ Halvorsen et al.^37^ found this correlation only in overweight (BMI > 25) and obese (BMI > 30) girls.

Exposure to sun as an aggravating factor was approximately three times less frequently reported than beneficial effect of both sun and artificial UVA (sunbeds) in the surveyed schoolchildren with acne. Controlled clinical trials on the therapeutic effect of sunlight in acne are lacking.^14^, ^38^ Short-term beneficial effects are due to tanning-related camouflage and the faster healing of inflammatory lesions caused by UV-induced erythema. However, long-term comedogenic (sebum squalene peroxidation) and carcinogenic effects are more evidence-based and clinically relevant.^39^

The influence of smoking was moderately frequently reported; 17.3% of boys *vs.* 11.4% girls recognized its ameliorating effects, which is not in accordance with the study by Rombouts et al.,^40^ where duration and extent of smoking was correlated with regression of papulopustular acne in girls only. Similarly, a French survey^41^ conducted on adolescents and young adults concluded that smoking more than ten cigarettes a day was highly associated with having no acne. Still, no relevant associations have been found by other authors.^42^, ^43^ Alcohol consumption had very low reported rate in the present study (5.1%), the same as observed in Portuguese adolescents,^44^ which is among lowest reported among similar studies.^13^, ^45^ The role of alcohol in the pathophysiology of acne is unclear, but a lifestyle which includes higher alcohol consumption might have some relation to acne, although not yet investigated.

Surprisingly high number of boys (58.5%) and girls (62.4%) in this study believed in the curative effect of cosmetic treatment, namely comedone extraction. This physical method is widely used, but the evidence of its efficacy in peer-reviewed journals is scarce.^46^ Recently, an increase in quality-of-life parameters has been detected in women (19–29 years) who have undergone some sort of cosmetic treatment for acne.^47^

Almost 30% of all participants believed that their acne got better when they spent a substantial time out of school, when on holidays. The authors hypothesize that the beneficial “holidays” effect is actually multifactorial, relying on factors such as decreased anxiety, a diet tending towards healthier servings, and avoidance of sleep disturbances.

“Cosmetics and make up” as an exacerbating factor among the surveyed girls herein was quite similarly reported in Korean acne sufferers, but was higher than reported beliefs in surveys conducted on both girls and boys.^9^, ^12^ The comedogenic properties of cosmetics are well supported; moreover, a cross-sectional study on 140 girls in Sri Lanka significantly correlated exposure to at least one cosmetic item and acne grade.^48^

Inadequate duration of sleep was reported to aggravate acne in 40.2% of Saudi males,^22^ and if proper, sleep was recorded to ameliorate acne in 32% of Greek schoolchildren.^9^ In the present study, acne triggering due to lack of sleep was determined in 10% of participants. Sleep disorders were not studied in terms of relationship with acne, except in a French study^49^ on a representative sample of adolescents and young adults where univariate analysis did not reveal any significant differences between the number of hours of sleep and the quality of sleep among the acne and control groups, but did determine a higher risk of difficulty falling asleep and feeling tired upon waking up among those with acne.

Premenstrual flare of acne has been usually studied in women with adult acne. In adolescent girls, Ghodsi et al.^24^ demonstrated that the premenstrual phase is an acne risk factor (*p* < 0.015); moreover, reports on beliefs show a frequency of 22–61% among girls with acne,^44^, ^45^ so the exceedingly high level reported in the present study (84.6%) warrants further research on adolescent girls in this region. Dermocosmetics are proven to be effective in amelioration of flare up.^50^

## Conclusion

Simple comparison between the perceptions of youths in this country and elsewhere regarding studies also based only on adolescents with acne,^5^, ^6^, ^8^, ^9^ dating back to 1983, objectively revealed some similarities as well as major disagreements in adolescents’ perspectives. However, it is believed that the concordance of estimated acne-related perceptions with objective literature data regarding specific factors that may influence acne aggravation or acne regression is more applicable for health care educational programs and reforms, and moreover, they could be implemented in an office-based dermatologist-oriented approach. Summarized recommendations for adolescents with acne according to the present research related to the supporting scientific literature are listed in Table 1.

**Table 1:** Summary of the most frequently reported acne-related beliefs in the present study and their concordance with evidence-based literature. Recommendations for proper behavior, where applicable.

Acne-related belief	Influence on acne in surveyed adolescents	Comment and evidence-based recommendation	Main reference
Sweating, exercise	Triggers	Individually based, NS	Poli F et al.,^19^ Short RW et al.^21^
Inadequate face wash	Triggers	Wash twice daily with cleanser	Choi JM et al.,^25^ Magin et al.^14^
Emotional stress	Triggers	Proven bidirectional relationship	Chiu A et al.,^16^ Yosipovitch G et al.^17^
Dietary change towards healthier foods	Improves	Recently supported	Burris JB et al.,^30^ Melnik et al.,^31^ Tan et al.^1^
Diet – sweets	Triggers	Dietary counseling	Burris JB et al.,^33^ Ghodsi et al.,^24^ Suh DH et Kwon HH^28^
Cosmetic treatment (comedo extraction)	Improves	Insufficient evidence – supports medical therapy	Taub AF,^46^ Chilicka K et al.^47^
Sun, UVA	Improves	NS comedogenesis and carcinogenesis	Zouboulis et al.,^38^ De Luca C et al.^39^
Drinking water	Improves	Not investigated elsewhere	
Premenstrual flare^a^	Triggers	Highly probable, dermocosmeceuticals efficient	Ghodsi et al.,^24^ Saint-Jean M^50^
Cosmetics^a^	Triggers	Avoid cosmetics when prone to acne	Perera MPN et al.^48^

a Girls only. NS, not supported (by literature data); N/A, not available.

To the authors’ knowledge, this is the first epidemiological study on representative sample in the Western Balkan region. The main advantage of the present study is the fact that the significance of acne triggering or ameliorating factors is presented from the perspective of available academic research and is focused on the perceived beliefs of those who are affected with the disease. Further efforts in whole region are needed to build a solid framework of investigative studies on adolescents with acne in the Western Balkans.

## Financial support

None declared.

## Author's contributions

Milica Markovic: Approval of the final version of the manuscript; conception and planning of the study; elaboration and writing of the manuscript; obtaining, analyzing and interpreting the data; effective participation in research orientation; intellectual participation in propaedeutic and/or therapeutic conduct of the cases studied; critical review of the literature.

Ivan Soldatovic: Statistical analysis; obtaining, analyzing and interpreting the data; critical review of the manuscript.

Milan Bjekic: Conception and planning of the study; obtaining, analyzing and interpreting the data; critical review of the literature.

Sandra Sipetic-Grujicic: Conception and planning of the study; critical review of the literature; critical review of the manuscript.

## Conflicts of interest

None declared.
